# Health care consequences of hospitalization with *Clostrioides difficile* infection: a propensity score matching study

**DOI:** 10.1186/s12879-022-07594-x

**Published:** 2022-07-15

**Authors:** Bruce E. Hirsch, Myia S. Williams, Dimitre G. Stefanov, Martin L. Lesser, Karalyn Pappas, Thomas Iglio, Craig Gordon, Renee Pekmezaris

**Affiliations:** 1 Division of Infectious Disease, Northwell Health, Manhasset, NY 11030 USA; 2 Donald and Barbara Zucker School of Medicine at Hofstra/Northwell, Hempstead, NY USA; 3 Division of Health Services Research, Department of Medicine, Northwell Health, Manhasset, NY 11030 USA; 4 Institute of Health Systems Science, The Feinstein Institutes of Medical Research, Manhasset, NY 11030 USA; 5 Biostatistics Unit, The Feinstein Institutes for Medical Research, Manhasset, NY 11030 USA; 6 Department of Planning, Northwell Health, New Hyde Park, NY 11042 USA

**Keywords:** *Clostridioides difficile* infection, Hospitalizations, SPARCS, Mortality

## Abstract

**Background:**

*Clostridiodies difficile* infection (CDI) has been characterized by the Center for Disease Control and Prevention (CDC) as an urgent public health threat and a major concern in hospital, outpatient and extended-care facilities worldwide.

**Methods:**

A retrospective cohort study of patients aged ≥ 18 hospitalized with CDI in New York State (NYS) between January 1, 2014–December 31, 2016. Data were extracted from NY Statewide Planning and Research Cooperative (SPARCS) and propensity score matching was performed to achieve comparability of the CDI (exposure) and non-CDI (non-exposure) groups. Of the 3,714,486 hospitalizations, 28,874 incidence CDI cases were successfully matched to 28,874 non-exposures.

**Results:**

The matched pairs comparison demonstrated that CDI cases were more likely to be readmitted to the hospital at 30 (28.26% vs. 19.46%), 60 (37.65% vs. 26.02%), 90 (42.93% vs. 30.43) and 120 days (46.47% vs. 33.74), had greater mortality rates at 7 (3.68% vs. 2.0%) and 180 days (20.54% vs. 11.96%), with significant increases in length of stay and total hospital charges (p < .001, respectively).

**Conclusions:**

CDI is associated with a large burden on patients and health care systems, significantly increasing hospital utilization, costs and mortality.

## Introduction

*Clostridiodies difficile* infection (CDI) has been characterized by the Center for Disease Control and Prevention (CDC) as an urgent public health threat [1] and a major concern in hospital, outpatient, and extended-care facilities worldwide [2–4]. The public health impacts of CDI are significant, with recent studies reporting annual healthcare costs to be as much as $4.8 billion for acute care facilities alone and a great deal of variation in “extra” length of stay (LOS) and associated costs [4, 5]. A 2015 review of CDI outcomes demonstrated that depending on the time frame- during endemic or epidemic periods of CDI, all-cause mortality and attributable mortality ranged from 11.8 to 38%, and 0–16.7% respectively [6, 7]. The burden of annual CDI incident infections in the US is estimated to be 453,000, cases per year, with increased incidence noted in those older than 65 years [8–10]. About 24% of cases were reported to occur in hospital settings, highlighting the need for the prevention of CDI [11]. The issue of recurring CDI is also of concern, with recurrence rates varying from 5 to 50%, with an average of 20% [8, 12]. Lessa et al. [8] estimated that about 83,000 first recurrent infections occur between 14 and 56 days after the initial episode. Such numbers are alarming, given the risk of transmission and challenges in treating recurrent infections [8, 12].

The purpose of our study was to investigate the impact of CDI on LOS, rehospitalization, mortality, and costs in patients hospitalized throughout New York State (NYS). We queried a large comprehensive, statewide database to assess the extent to which CDI patients have higher rehospitalization rates, greater health care expenditures, and mortality than matched controls without CDI. Gaining a better understanding of the extended natural history of CDI may inform the need for interventions to enhance clinical outcomes and public health. Furthermore, the focus on in-patients assures greater uniformity of diagnosis and management. It is the acute care setting that integrates the epidemiologic concerns of clinical severity and patient vulnerability with the extended natural history of CDI as measured by the consequential- if crude- rehospitalization and mortality rates. Our study complements and broadens previous studies with a longer-term follow-up of individuals compared with matched controls.

## Methods

### Data sources

A retrospective cohort study of patients hospitalized in NYS was conducted. The Feinstein Institutes of Medical Research Institutional Review Board deemed that the study did not meet the definition of human subjects research; therefore, IRB review was not needed. Data were extracted from the New York Statewide Planning and Research Cooperative (SPARCS)and cross-referenced by investigators with death data from Vital Statistics of New York State, to identify deaths that occurred *after* hospital discharge. All protocol methods and use of this data were carried out in accordance with relevant guidelines and regulations.

SPARCS is a longitudinal comprehensive all-payer data reporting system created in 1979 to collect information on hospital discharges from all Article 28 facilities operating in NYS. SPARCS is one of the largest administrative data systems in the country, with over 2.3 million annual inpatient discharges and 6.7 million treat and release (i.e., same day) emergency room visits [13]. SPARCS currently collects patient-level detail on patient characteristics, diagnoses, and treatments based on ICD-9/10 codes, services, and charges/insurance claims for each hospital inpatient stay and outpatient visit [14]. Death data were accessed from the New York State Vital Statistics Program, which registers live births, deaths, fetal deaths, induced terminations of pregnancy/abortions, etc.

### Study population and selection criteria

All patients aged ≥ 18 years hospitalized between January 1, 2014–December 31, 2016 were included in the analysis. A known CDI exposure was defined as one of the ICD-9 and ICD-10 principal diagnostic codes: 00845, A0472, and A047. There were 3,714,486 total hospital discharges in NYS that met the eligibility criteria during the 2-year interval, of which 28,897 had a de novo CDI diagnosis (accounting for 0.78% of all discharges) and 3,685,589 did not. The total sample of 3,714,486 was as a result of removing 23 hospital discharges (of which one was a CDI diagnosis) due to missing gender. Final propensity score matching (PSM) was 28,874 incident CDI exposures successfully matched to 28,874 non-exposures.

### Exposure of interest

The exposure of interest was diagnosis of CDI during hospitalization as documented in the medical chart. The data set was comprised of discharges between 01/01/14 and 12/31/16, the primary period of analysis corresponded to 07/01/2014–06/30/2016. There was a 6-month look-back period (01/01/2014–06/30/2014) to determine whether a diagnosis of CDI in the analysis period was de novo (incident case) or a recurrent case. A 6-month follow-up period (07/01/2016–12/31/2016) served to determine whether an exposed or control subject had any subsequent hospitalizations. The use of a 6-month look-back period represented an attempt to completely exclude recurrent cases stricter than the CDC definition for recurrence to enhance our confidence in identifying exposures and non-exposures. It is important to note that we did not capture outpatient diagnoses of CDI as well as prior inpatient CDI diagnoses during the look back period. De novo CDI exposures were defined as an exposure of CDI in period of analysis, provided that there was no CDI visit for that patient in the look-back period, to avoid capture of recurrent infection. Non-exposures were defined as all visits from subjects that never had a CDI diagnosis. Exposures and non-exposures who died in the hospital during the first visit were excluded from the analysis. In the event that an exposure or non-exposure died on the second or subsequent hospital visit, all data prior to that visit were included from the analysis.

### Variables and outcomes

The primary outcome in this study was 30-day readmission. Secondary outcomes included 60, 90, 120, 180 days readmissions, mortality within 7, 15, 30, 180 and 360 days of discharge, hospital LOS and total charges. Secondary outcomes were analyzed 7, 15, 30, 90, 180, and 360 days from the date of discharge from the index hospitalization as long as they occurred within the 6-month follow-up period. Age, race, comorbidities (Charlson comorbidity index), insurance status, gender, and ethnicity.

### Propensity score matching (PSM)

In this retrospective cohort study, comparability of the CDI (exposure) and non-CDI (non-exposure) groups regarding potential confounding variables. This was accomplished using PSM. The propensity scores were generated using a logistic regression model from the following variables: age, gender, Charlson Comorbidity Index (CCI), race, ethnicity, insurance type, and month/year of admission. PSM was accomplished using greedy nearest neighbor 1:1 matching based on logit (PS) with a caliper of 0.05, with an exact matching on month/year of admission.

### Statistical analyses

There were a total of 3,714,486 hospitalizations in New York State that met the eligibility criteria in the 2-year interval between 7/1/14 and 6/30/16, of which 28,897 were identified as having CDI. Of these, 28,874 incident CDI exposure were successfully matched to 28,874 non-exposures Hospitalization that met eligibility criteria was identified and incident CDI exposures were determined. All exposures, with the exception of the 23 instances of incomplete data, were successfully matched. The maximum standardized difference as a percentage was 1.7% (Table 1), which is below the suggested upper limit of 10% [15, 16] and indicated a good balance between the matched groups. The variance ratios between the CDI and the pure non-exposures groups were between 1 and 1.03 for all variables in the matched observations, which is within the recommended range of 0.5 to 2 [17].

**Table 1:** Baseline characteristics of the incident CDI and control groups in the propensity score matched sample

Variable	Incidence CDI cases N = 28,874	Controls N = 28,874	Standardized difference in %^#^	Variance ratio
Age	67.55 ± 17.73 [median 70 (57–81)]	67.67 ± 17.63 [median 70 (57–81)]	− 0.626	1.01
Female sex	57.42%	57.34%	0.168	1.00
Race				
White	68.94%	69.17%	− 0.493	1.00
Black	13.92%	13.71%	0.573	1.01
Other	17.12%	17.14%	0.061	1.00
Ethnicity_class Hispanic/other	11.51%	11.38%	0.362	1.01
Insurance_status				
Private/other	20.62%	20.60%	0.056	1.00
Medicare	65.20%	65.47%	− 0.557	1.00
Medicaid	14.18%	13.93%	0.618	1.01
Total CCI groups per record**	1.12 ± 1.43 [median 0 (0–2)]	1.10 ± 1.41 [median 0 (0–2)]	1.707	1.03

All categorical data are reported as percentages. The 2 continuous variables, Age and ‘Total CCI groups per record’ are reported as mean ± SD and median (25th,75th percentiles)

Baseline characteristics of the incident CDI exposure and non-exposure groups were compared using chi-square and two-sample t-tests, as appropriate (Table 2). Comparisons between the matched CDI exposures and non-exposures groups were based on McNemar’s test (mortality and the readmission indicators), paired t-test (Log (total charges)) and Wilcoxon signed-rank test (LOS). Since patients were clustered within hospitals, we performed a sensitivity analysis for the readmission and mortality outcomes, accounting for such clustering. We used generalized linear mixed models, with a random effect for the hospital and a random effect for the matched pair. All categorical data are reported as percentages. Continuous variables are reported as mean ± SD and median (25th, 75th percentiles). Summary statistics for the outcomes, with the 95% confidence intervals (CI) for the difference between the matched groups are presented (Table 3). A result was considered statistically significant at the p < .05 level. All analyses were performed using SAS version 9.4 (SAS Institute Inc, Cary, NC).

**Table 2:** Baseline characteristics of the incident CDI and control groups

Variable	Incidence CDI cases N = 28,897	Controls N = 3,685,589	Standardized difference in %^#^	p-value*
Age	67.55 ± 17.72 [median 70 (57–81)]	56.69 ± 20.79 [median 58 (38–73)]	56.182	< 0.001
Female sex	57.41%	57.59%	− 0.365	0.5365
Race				< 0.001
White	68.91%	58.88%	21.012	
Black	13.93%	18.65%	− 12.833	
Other	17.16%	22.47%	− 13.345	
Ethnicity_class Hispanic/Other	11.52%	16.18%	− 13.499	< 0.001
Insurance_status				< 0.001
Private/other	20.61%	31.58%	− 25.184	
Medicare	65.19%	41.95%	47.919	
Medicaid	14.20%	26.46%	− 30.842	
Total CCI groups per record**	1.12 ± 1.43 [median 0 (0–2)]	0.74 ± 1.20 [median 0 (0–1)]	29.239	< 0.001

*P-values based on the Chi-square and the two-sample t-tests

^#^The standardized difference in% is the mean difference as a percentage of the average standard deviation

All categorical data are reported as percentages. The 2 continuous variables, Age and Total CCI groups per record are reported as mean ± SD and median (25th, 75th percentiles)

**Table 3:** Analysis of the primary and the secondary outcomes

Outcome	Incidence CDI cases	Control	Diff (95% CI)	p-value*	OR(95% CI)**
30 d Readmission (n = 28,846 pairs)	28.26%	19.46%	8.81 (8.11–9.5)	< 0.0001	1.63 (95% CI 1.57–1.70)
60 d Readmission (n = 28,846 pairs)	37.65%	26.02%	11.62 (10.87–12.38)	< 0.0001	1.71(95% CI 1.65–1.77)
90 d Readmission (n = 28,846 pairs)	42.93%	30.43%	12.5 (11.72–13.28)	< 0.0001	1.71(95% CI 1.66–1.77)
120 d Readmission (n = 28,846 pairs)	46.47%	33.74%	12.73 (11.94–13.52)	< 0.0001	1.70 (95% CI 1.64–1.76)
180 d Readmission (n = 28,846 pairs)	51.39%	38.76%	12.63 (11.82–13.43)	< 0.0001	1.66 (95% CI 1.61–1.72)
Mortality within 7 days of discharge (n = 28,874 pairs)	3.68%	2.01%	1.67 (1.4–1.94)	< 0.0001	1.86 (95% CI 1.68–2.07)
Mortality within 15 days of discharge (n = 28,874 pairs)	5.76%	3.17%	2.59 (2.26–2.93)	< 0.0001	1.87 (95% CI 1.72–2.03)
Mortality within 30 days of discharge (n = 28,874 pairs)	8.65%	4.71%	3.93 (3.53–4.34)	< 0.0001	1.90 (95% CI 1.78–2.03)
Mortality within 180 days of discharge (n = 28,874 pairs)	20.54%	11.96%	8.58 (7.98–9.17)	< 0.0001	1.88 (95% CI 1.80–1.97)
Mortality within 360 days of discharge (n = 28,874 pairs)	26.15%	16.55%	9.6 (8.93–10.26)	< 0.0001	1.75 (95% CI 1.69–1.82)
LOS in days-Median (25-75th Perc.) (n = 28,874 pairs)	9 (5–16)	4 (2–7)	4 (0–11)	< 0.0001	
Total Charges ($)- Geometric mean (95% CI) (n = 28,874 pairs)	58,430 (57,689 − 59,181)	31,705 (31,363 − 32,051)	Percentage increase of total charges for CDI relative to control: 84% (81–87%)	< 0.0001	

*p-values were based on the McNemar’s test (Mortality and the Readmission indicators), the paired t-test (Log(Total Charges)) and the Wilcoxon signed rank test (LOS)

**p-values based on a generalized linear mixed model with a random effect for hospital and a random effect for the matched pair

## Results

There were 3,714,486 total hospital discharges in NYS that met the eligibility criteria during the 2-year interval, of which 28,897 (0.78%) had a de novo CDI diagnosis.

### Readmission

Matched pairs comparison of the 28,874 incident CDI exposures to 28,874 non-exposures demonstrated that CDI exposures were more likely to be readmitted to the hospital at all time points assessed. Hospital readmission rate at 30 days was 28.26% for CDI exposures versus 19.46% for non-exposures (p < .0001, absolute difference = 8.81%, Table 3). Similarly, the hospital readmission rate at 60 days was 37.65% for CDI exposures, versus 26.02% for non-exposures (p < .0001, absolute difference = 11.62%). The hospital readmission rate at 90 days was 42.93% for CDI exposures, versus 30.43% for non-exposures (p < .0001, absolute difference = 12.50%). The hospital readmission rate at 120 days was 46.47% for CDI exposures versus 33.74% for non-exposures (p < .0001, absolute difference = 12.73%). Finally, the hospital readmission rate at 180 days was 51.39% for CDI exposures versus 38.76% for non-exposures (p < .0001, absolute difference = 12.63%).

### Mortality

We found greater mortality rates in CDI exposures at all-time follow-up intervals (Table 3). The mortality rate at 7 days was 3.68% for CDI exposures versus 2.01% for non-exposures (p < .0001, 1.8-fold increase, absolute difference = 1.67%). The mortality rate at 180 days was 20.54% for CDI exposures versus 11.96% for non-controls (p < .0001, 1.72-fold increase, absolute difference = 8.58%).

The results of the sensitivity analysis, accounting for clustering within hospitals, were nearly identical to the presented results for the readmission and mortality outcomes and are not included in the manuscript.

### Length of stay

LOS was significantly longer (p < .0001) in the CDI matched group. We used the Wilcoxon signed-rank test to compare the distribution of this outcome in the two groups, due to its skewness. Median LOS in days (25–75th percentile) was 9 (5–16) 4 (2–7) for the CDI exposure and non-exposure matched groups (Table 3).

### Total charges

Hospital charges were significantly greater in CDI exposures (p < .0001). The median (25–75th percentile) hospital charges were $55,171 ($26,753–$119,933) and $30,811 ($16,693–$57,838) (Table 3) for the CDI exposures and non-exposures matched groups, respectively, an increase of an additional $24,360. We compared total charges after log transformation, due to the skewed distribution of this outcome. The geometric mean (95% CI) for the total charges were estimated to be $58,430  ($57,689–$59,181) and $31,705 ($31,363–$32,051) in the CDI and control groups, respectively. On average, the total charges in the CDI exposure group were 1.84 times (95% CI 1.81 to 1.87, p < .0001) higher than the matched non-exposure group.

## Discussion

A number of studies have investigated the impact of CDI, however, there has been a dearth of *large-scale* investigations that have statistically accounted for important confounding variables such as the severity of illness, age, and insurance status. Our study was able to match 28,874 incident CDI exposures to 28,874 non-exposures, accounting for important confounding variables to give us a comprehensive understanding of the impact of CDI. The objective of our study was to investigate the impact of de novo CDI in hospitalized patients upon re-hospitalization, costs, and mortality in NYS.

In our PSM study, utilizing the NYS SPARCS database of hospitalizations cross-referenced with mortality data from Vital Statistics of New York State, we found that hospitalization with CDI is common, occurring in 28,897 discharges during the 2-year period (7/1/14 and 6/30/16), accounting for 0.78% of all hospitalizations. When compared with non-exposures, our data documented that enhanced rates of readmission were sustained at 30, 60, 90, and 120 days. The maintenance of the absolute difference throughout these periods suggests that CDI cases are distinguished by factors other than simply the experience of CDI.

Our data also indicates greater mortality rates in CDI cases at all time intervals compared to controls: at 7, 15, 30, 180, and 360 days of discharge- (p < .0001). This enhanced mortality rate was also sustained through all examined follow-up intervals. Greater utilization of health care resources was documented in CDI cases as well: in terms of costs, our study found that the total charges were 84% higher in the CDI group compared to the non-exposed group. In NYS alone, we found these inpatient charges approached a billion dollars during the two-year study period. Of note, charge data (the dollar amount a health care provider sets for services rendered) can be different from the amount the provider is paid (cost). It may well be that the CDI is a marker of vulnerability, as enhanced mortality and the need for additional health care resources experienced by patients hospitalized with CDI are maintained throughout the follow-up period.

Our comprehensive evaluation confirms previous work describing enhanced rehospitalization rates [6, 18]. The current study contributes to the quantification of the enhanced readmission rate, mortality rate, and cost of care with prolonged follow-up. We found that CDI is associated with an increased median length of stay of 5 days and an additional $27,891 in hospital charges per admission. Our data indicate an increase in readmission rates of approximately 30% at 30, 60, 90, and 120 days of follow-up. We were surprised to note that hospitalization with CDI was associated with a doubling of mortality through 30 days, with a sustained increased risk, which only modestly decreased at 360 days. Our finding that hospitalization with CDI is a marker for major adverse outcomes sustained through 360 days indicates additional characteristics of the epidemiology of CDI to explore and informs the computation of benefits of interventions.

Our study has several limitations. While the New York State SPARCS system is a comprehensive database of acute hospitalizations, there are multiple limitations. The reliance on ICD-9 and ICD-10 codes introduces potential inaccuracies in identifying CDI cases [14, 19, 20]. Furthermore, our data set corresponds with the timeline within which the conversion from ICD-9 to ICD-10 occurred. In clinical practice, CDI is diagnosed with laboratory testing with symptomatic diarrhea. We and others have identified circumstances in which indiscriminate testing may identify mere colonization instead of active infection [19, 21]. One study that used a population-based dataset to determine the effect of using ICD versus laboratory CDI diagnosis found ICD codes provided similar epidemiological time trend patterns as laboratory data in identifying CDI [21]. Thus, even with the absence of clinical detail ICD codes are reliable to allow the identification of a cohort of hospitalized patients with at least colonization with the organism [19, 21].

In addition to the potential misclassification from using codes, the timing of CDI is crucial for accurate estimation of the attributable length of stay and charges [22]. Unfortunately, the available data does not allow us to determine the timing, severity or to distinguish between community-acquired versus hospital acquired cases and explore potential confounding variables such as specific medical diagnoses, antibiotics exposure, nutritional or functional status [14, 19]. Attributing all costs and days to a time-dependent event leads to time-dependent bias and over-estimation of attributable charges/LOS [22].

The exclusion of patients who died within the first hospitalization biased the charges and LOS analysis. This created a selection and downward bias. In our analysis, we excluded these patients because, in order to evaluate the primary outcome, which was 30-day re-admission, the patient had to be discharged from their first hospitalization to be at risk for re-admission. While the exclusion of these patients biases the first hospitalization and LOS downward, it is a necessary limitation of the analysis.

Our findings are that hospitalization with CDI is a marker with consequential health care ramifications for future research, clinical management, and health prevention. The increased hospital readmission rate, mortality, and health care costs associated with hospitalization for CDI poses a significant public health challenge. The gut microbiome represents a crucial therapeutic target for patients with CDI [16–18]. Prevention strategies including antibiotic stewardship and conservatorship of the gut microbiome on long-term health outcomes warrants investigation.

## Data Availability

The data that support the findings of this study are available from New York State Department of Health (NYSDOH) but restrictions apply to the availability of these data, which were used under license for the current study, and so are not publicly available. Data are however available from the New York State Department of Health subject to their Data Protection Review Board. Please contact the corresponding author @mwilliam26@northwell.edu for data.
